# Complement and immunoglobulin profiles in ANCA-associated vasculitis with renal involvement: a serotype-stratified analysis of disease activity and renal outcomes

**DOI:** 10.3389/fimmu.2026.1912981

**Published:** 2026-10-06

**Authors:** Sonja Golubović, Vladimir Veselinov, Vladimir Đurović, Milica Popović, Violeta Knežević

**Affiliations:** 1 Faculty of Medicine, University of Novi Sad, Novi Sad, Serbia; 2 Clinic for Nephrology and Clinical Immunology, University Clinical Center of Vojvodina, Novi Sad, Serbia

**Keywords:** ANCA-associated vasculitis, complement, immune phenotyping, immunoglobulins, MPO-ANCA, PR3-ANCA, renal outcomes, serotype

## Abstract

**Background:**

ANCA-associated vasculitis (AAV) is characterized by pauci-immune small-vessel inflammation driven predominantly by neutrophil activation, in contrast to immune-complex–mediated glomerulopathies in which complement consumption tracks disease activity and renal injury severity. Serum C3 at diagnosis has repeatedly been associated with renal and patient survival in AAV, yet it remains unresolved whether complement fractions reflect the extent of renal injury in a dose-dependent manner, as they do in lupus nephritis, and how complement relates to immunoglobulin class profiles across ANCA serotypes.

**Methods:**

This retrospective single-center cohort study included 73 patients with AAV (PR3-ANCA, n = 39; MPO-ANCA, n = 34) who had complete complement and immunoglobulin data at admission and at least 6 months of follow-up at a tertiary nephrology center. Serum C3, C4, IgG, IgA, and IgM were measured at baseline before the initiation of immunosuppressive treatment; no sample was obtained after plasma exchange had begun. Patients were stratified by ANCA serotype and chronic kidney disease (CKD) severity. Associations with disease activity, inflammatory indices, and 6-month outcomes were explored using non-parametric comparisons, Spearman correlations, false discovery rate (FDR) correction, and sensitivity analyses.

**Results:**

C3 and C4 lay within the reference interval in 97.3% and 86.3% of patients, respectively, and showed no gradient across CKD severity groups (C3 p = 0.453; C4 p = 0.550), whereas the inflammatory indices C-NLR and PLR differed across those groups. IgA was nominally higher in patients with MPO-ANCA than in those with PR3-ANCA (2.60 [1.92–2.88] vs. 2.11 [1.78–2.30] g/L, p = 0.011) but did not retain significance after FDR adjustment across the five immune parameters (q = 0.055), and all values lay within the reference interval. IgG correlated with ESR (ρ = +0.40, p < 0.001) and ΔeGFR at 6 months (ρ = +0.26, p = 0.037). Serum C4 was higher in the eight patients who did not achieve clinical remission (0.35 [0.28–0.44] vs. 0.28 [0.20–0.33] g/L, nominal p = 0.037; FDR q = 0.184; AUC 0.729, 95% bootstrap CI 0.473–0.953, an interval that includes 0.5). C3 correlated inversely with neutrophil-to-lymphocyte ratio (NLR) (ρ = −0.24, p = 0.043).

**Conclusions:**

Frank complement consumption was uncommon in this cohort, and neither C3 nor C4 graded renal injury severity, consistent with pauci-immune pathogenesis and in contrast to lupus nephritis. These data do not conflict with published cohorts in which relatively lower C3 predicted adverse renal outcomes; rather, they suggest that the prognostic signal resides in variation within the reference interval rather than in measurable consumption proportional to glomerular injury. The serotype difference in IgA and the association between C4 and failure to achieve remission are exploratory observations that did not withstand correction for multiple comparisons. Because induction-regimen coding was sparse and heterogeneous, no treatment-adjusted IgA model was retained. The C4 association was based on only eight non-remission events and, notably, ran in the opposite direction to a complement-consumption interpretation; it requires confirmation in larger, prospective cohorts.

## 1 Introduction

ANCA-associated vasculitis (AAV) encompasses a group of necrotizing small-vessel vasculitides defined by circulating antineutrophil cytoplasmic antibodies (ANCA), with granulomatosis with polyangiitis (GPA) and microscopic polyangiitis (MPA) representing the principal clinical phenotypes predominantly associated with PR3-ANCA and MPO-ANCA, respectively (1). Renal involvement, occurring in up to 80% of patients, is a major determinant of morbidity and a leading driver of progression to end-stage kidney disease despite modern immunosuppressive regimens (2, 3).

The immunopathogenesis of AAV is driven predominantly by neutrophil activation, neutrophil extracellular trap (NET) formation, and direct endothelial injury rather than by immune complex deposition (4). This pauci-immune mechanism stands in contrast to lupus nephritis (LN), in which immune complex–mediated complement activation results in measurable consumption of C3 and C4 that correlates closely with disease activity and renal injury severity (5, 6). In LN, depressed complement fractions serve as established clinical biomarkers of flare and guide treatment response monitoring.

The relationship between systemic complement and renal disease in AAV has, in fact, been examined in several cohorts, with consistent findings. Augusto et al. reported that although serum C3 remained within the reference interval across their entire cohort, patients below the cohort median had significantly poorer patient and death-censored renal survival, and lower C3 was associated with a higher proportion of glomeruli showing cellular crescents (7). Crnogorac et al., in 75 patients with renal AAV, found that C3 below 0.90 g/L at diagnosis predicted end-stage kidney disease and death (8), and comparable associations have been described by Villacorta et al. (9) and, in 139 immunosuppression-naïve patients, by Choi et al. (10). What remains unresolved is the nature of this signal: whether it reflects graded complement consumption proportional to the extent of glomerular injury, as in LN, or a distinct phenomenon operating largely within the reference interval. Distinguishing between these possibilities has direct practical consequences for whether C3 and C4 can be used as markers of renal injury severity in routine care.

Complement activation does occur in AAV, particularly through the alternative pathway (11, 12), and the therapeutic efficacy of avacopan—a selective C5a receptor antagonist—confirms the biological relevance of this pathway (13, 14). However, locally amplified complement signaling at the tissue level may not translate into measurable systemic complement consumption under routine clinical conditions. The selective alternative pathway involvement in MPA, contrasted with NET-mediated and adaptive immune mechanisms predominating in GPA/PR3-ANCA disease, raises the hypothesis that complement and immunoglobulin profiles may differ between serotypes and carry distinct prognostic information.

Immunoglobulin classes have received limited systematic attention as biomarkers in AAV. IgG hypergammaglobulinaemia may reflect chronic polyclonal B-cell stimulation in active systemic vasculitis, IgA has been associated with mucosal immune dysregulation in granulomatous disease, and IgM changes may reflect early B-cell responses. Where serotype-specific humoral differences have been demonstrated in AAV, they have generally been qualitative rather than quantitative—differences in IgG Fc galactosylation and sialylation, for example, distinguish MPO-ANCA disease from PR3-ANCA disease and track activity differently between serotypes (15). Whether total immunoglobulin class concentrations, which are measured routinely and inexpensively, carry any comparable information has not been established.

The present study therefore aimed to (1) characterize serum C3, C4, IgG, IgA, and IgM across PR3-ANCA and MPO-ANCA serotypes; (2) determine whether complement fractions grade renal injury severity across chronic kidney disease (CKD) stages, as would be expected if consumption were proportional to glomerular injury; (3) assess correlations between these immune parameters and established disease activity scores and renal function; and (4) explore their association with clinical remission and renal recovery at 6 months. Given the sample size and the number of comparisons, objectives (3) and (4) are exploratory and hypothesis-generating.

## 2 Materials and methods

### 2.1 Study design and patient population

This retrospective single-center cohort study was conducted at the Clinic for Nephrology and Clinical Immunology, University Clinical Center of Vojvodina, Novi Sad, Serbia. Consecutive patients with confirmed AAV admitted between 2020 and 2023 were eligible if they had (1) confirmed PR3-ANCA or MPO-ANCA positivity on ELISA; (2) complete serum C3, C4, and immunoglobulin class data at admission; and (3) a minimum of 6 months of follow-up. Diagnosis was established according to the 2022 ACR/EULAR classification criteria for GPA and MPA (16, 17). Patients with concurrent active malignancy, known primary immunodeficiency, or other systemic autoimmune diseases associated with secondary immunoglobulin abnormalities were excluded. Of 88 patients in the institutional AAV registry, 73 had complete complement and immunoglobulin data and constituted the final analytic cohort. The 15 excluded patients did not differ significantly from the study cohort in age, sex, or baseline eGFR (all p > 0.20).

### 2.2 Ethics statement

The study was conducted in accordance with the Declaration of Helsinki and approved by the Institutional Review Board of the University Clinical Center of Vojvodina (protocol code 00-88, approval date 21 April 2023). Individual informed consent was waived given the retrospective design and the use of anonymized data only.

### 2.3 Laboratory measurements and timing of sampling

Complement fractions (C3 and C4) and immunoglobulin classes (IgG, IgA, and IgM) were measured by nephelometry at the central laboratory of the University Clinical Center of Vojvodina. The reference intervals applied were: C3 0.90–1.80 g/L, C4 0.16–0.38 g/L, IgG 7.0–16.0 g/L, IgA 0.7–4.0 g/L, IgM 0.4–2.3 g/L.

All complement and immunoglobulin measurements were obtained from the baseline admission laboratory panel before initiation of immunosuppressive treatment. No sample was obtained after plasma exchange had been initiated. Thus, all C3, C4, IgG, IgA, and IgM measurements included in the present analysis represent pretreatment values.

Inflammatory indices were calculated from the admission complete blood count: neutrophil-to-lymphocyte ratio (NLR) = absolute neutrophil count/absolute lymphocyte count; platelet-to-lymphocyte ratio (PLR) = platelet count/absolute lymphocyte count; C-reactive protein (CRP)-to-lymphocyte ratio (C-NLR) = CRP (mg/L)/absolute lymphocyte count (× 10^9^/L). Estimated GFR was calculated using the 2021 CKD-EPI creatinine equation (18). Disease activity was quantified using the Birmingham Vasculitis Activity Score (BVAS) version 3 (19) and the Five-Factor Score (FFS) (20). ANCA specificity was determined by ELISA.

### 2.4 Clinical phenotype and treatment data

Extrarenal phenotype data were abstracted retrospectively from the medical records where they could be retrieved reliably. Pulmonary and ear–nose–throat (ENT) involvement were sufficiently documented for analysis. ENT involvement was coded as present when the treating physician’s contemporaneous record explicitly documented upper-airway/ENT involvement attributable to AAV. Epistaxis and sinusitis were separately extractable manifestations and are reported individually, but they were not intended to exhaust the composite ENT category. Other extrarenal manifestations could not be reconstructed reliably and were therefore not included in comparative analyses.

Treatment data available in the source database included intravenous methylprednisolone pulse use, cyclophosphamide-based induction, rituximab-based induction, plasma exchange, and acute haemodialysis. Small numbers of mycophenolate, azathioprine, and methotrexate entries could not be classified reliably as induction versus maintenance treatment and were therefore not assigned to either category.

### 2.5 Patient stratification

Patients were stratified by ANCA serotype (PR3-ANCA vs. MPO-ANCA) and by CKD stage at admission based on eGFR: severely impaired (<15 mL/min/1.73 m², G5; n = 27), moderately to severely impaired (15–29 mL/min/1.73 m², G4; n = 15), and mildly to moderately impaired or preserved (≥30 mL/min/1.73 m², G3 or better; n = 31).

### 2.6 Outcome definitions

Clinical remission at 6 months was defined as BVAS = 0 with stable or improving renal function. Renal recovery was quantified as ΔeGFR (eGFR at 6 months minus eGFR at admission). Patients who died during follow-up were classified as non-remission cases. Baseline serotype, complement/immunoglobulin, pulmonary, and ENT variables used in the principal cross-sectional analyses were available for all 73 patients. Six-month remission status was available for 70/73 patients (39/39 PR3-ANCA and 31/34 MPO-ANCA); the three patients without evaluable 6-month outcome data were therefore excluded only from remission analyses.

### 2.7 Statistical analysis

Continuous variables are presented as median [interquartile range (IQR)]. Between-group comparisons used Mann–Whitney U tests (two groups) and Kruskal–Wallis tests with *post-hoc* pairwise Mann–Whitney U comparisons (three groups); categorical variables were compared using Fisher’s exact test. Spearman’s rank correlation coefficient (ρ) assessed associations between parameters. Statistical significance was set at p < 0.05 (two-tailed). Analyses were performed in Python 3.12 (SciPy 1.11, scikit-learn 1.5, statsmodels 0.14).

#### 2.7.1 Multiple comparisons

Five immune parameters (C3, C4, IgG, IgA, and IgM) were compared within each of three prespecified families of comparisons: by ANCA serotype, by CKD severity group, and by remission status. Within each family, nominal p-values were adjusted using the Benjamini–Hochberg procedure to control the false discovery rate (FDR) at 5% (21); both nominal p and FDR-adjusted q-values are reported. The correlation analyses (**Table 1**) involve a larger number of tests and are reported without adjustment; they are explicitly framed as exploratory and hypothesis-generating, and no inference of independent or incremental predictive value is drawn from them.

**Table 1:** Spearman rank correlations between complement/immunoglobulin parameters and clinical variables (exploratory, unadjusted, not corrected for multiple testing).

Parameter	BVAS	eGFR_0_	eGFR_6_	ΔeGFR	CRP	ESR	NLR	PLR	C-NLR
C3	ns	ns	ns	ns	ns	ns	−0.24 *	ns	ns
C4	ns	ns	−0.25 *	ns	ns	ns	ns	ns	ns
IgG	ns	ns	ns	+0.26 *	+0.26 *	+0.40 ***	ns	+0.24 *	ns
IgA	ns	ns	ns	ns	ns	+0.31 **	ns	ns	ns
IgM	ns	ns	ns	+0.25 *	+0.37 **	ns	ns	ns	+0.30 *

Spearman ρ shown where nominally significant; all other cells ns. *p < 0.05; **p < 0.01; ***p < 0.001. ΔeGFR, eGFR at 6 months minus eGFR at admission. These correlations are exploratory and are not corrected for multiple comparisons; no inference of independent or incremental predictive value is drawn from them.

#### 2.7.2 Partial correlations

Partial Spearman correlations were computed by rank-transforming both variables, regressing each on the covariate set by ordinary least squares, and taking the Pearson correlation of the residuals as the partial rank correlation coefficient (ρ_partial). The p values were derived from the corresponding t statistic with n − 2 − k degrees of freedom, where k is the number of covariates. The covariate sets are stated with each result.

Adjusted analysis of the serotype difference in IgA. We did not retain a multivariable-adjusted IgA model in the final analysis. Induction treatment could not be coded with sufficient granularity for a stable covariate: cyclophosphamide accounted for most recorded induction therapy, rituximab was recorded in only one patient, and several other immunosuppressant entries could not be classified reliably as induction versus maintenance. Under these conditions, treatment adjustment would create a sparse and potentially influential category and could give a misleading impression of precision. The serotype comparison of IgA is therefore presented as an unadjusted, FDR-corrected exploratory analysis only.

#### 2.7.3 Outcome analyses

Only eight non-remission events occurred. Multivariable modelling of remission was therefore not statistically defensible, and we deliberately refrain from presenting treatment-adjusted effect estimates that the data cannot support. Instead, the C4–remission association is presented unadjusted, together with (i) a partial correlation adjusted for ANCA serotype alone, (ii) a sensitivity analysis excluding patients exposed to plasma exchange, and (iii) a sensitivity analysis restricted to patients receiving cyclophosphamide-based induction. Receiver operating characteristic (ROC) analysis used Youden’s J with 2,000-iteration percentile-bootstrap 95% confidence intervals and is presented descriptively only. With only eight non-remission events, both the bootstrap interval and the data-derived Youden threshold are unstable. Any discordance between the ROC interval and the Mann–Whitney test is interpreted as a small-sample artefact rather than as evidence of a robust discriminative effect.

Generative AI was used for language editing and editorial assistance only. No generative AI tools were used for data collection, statistical analysis, or interpretation. The authors reviewed and take full responsibility for the final content.

## 3 Results

### 3.1 Patient characteristics, clinical phenotype and treatment

In all, 73 patients with AAV were included (PR3-ANCA n = 39, 53%; MPO-ANCA n = 34, 47%). Median age was 60.0 [51.0–67.0] years. MPO-ANCA patients were significantly older (65.5 [59.3–69.0] vs. 57.0 [49.0–61.5] years, p = 0.001). PR3-ANCA patients had higher FFS (2.0 [1.0–2.0] vs. 1.0 [0.3–2.0], p = 0.015) and higher rates of plasma exchange (33% vs. 9%, p = 0.021). NLR, PLR, and C-NLR were significantly higher in PR3-ANCA patients (all p ≤ 0.030).

Extrarenal phenotype data that could be reliably retrieved from the retrospective records are reported in **Table 2**. Pulmonary involvement was present in 46/73 patients (63%), with no significant difference between serotypes. ENT involvement was more frequent in PR3-ANCA than MPO-ANCA disease (32/39 [82.1%] vs. 17/34 [50.0%], Fisher p = 0.006). ENT involvement was a composite based on explicit physician documentation of AAV-related ENT disease; epistaxis (3/39 [7.7%] vs. 2/34 [5.9%]) and sinusitis (13/39 [33.3%] vs. 10/34 [29.4%]) were separately extractable manifestations and therefore do not sum to the composite ENT count. Other extrarenal manifestations could not be reconstructed reliably from the available retrospective records. Six-month remission status was available for 70/73 patients: all 39 PR3-ANCA patients and 31/34 MPO-ANCA patients.

**Table 2:** Baseline characteristics, organ involvement, and treatment stratified by ANCA serotype (n = 73).

Variable	Total (n = 73)	PR3-ANCA (n = 39)	MPO-ANCA (n = 34)	p
Age (years)	60.0 [51.0–67.0]	57.0 [49.0–61.5]	65.5 [59.3–69.0]	0.001 ***
Sex, male	43 (59%)	23 (59%)	20 (59%)	1.000 ns
eGFR at admission (mL/min/1.73 m²)	22.8 [12.0–46.0]	16.0 [12.0–37.0]	30.5 [12.0–56.8]	0.261 ns
Creatinine at admission (μmol/L)	316 [134–451]	357 [222–464]	211 [104–443]	0.140 ns
BVAS	15.0 [12.0–20.0]	17.0 [14.0–21.0]	13.0 [12.0–18.8]	0.069 ns
FFS	1.0 [1.0–2.0]	2.0 [1.0–2.0]	1.0 [0.3–2.0]	0.015 *
NLR	4.2 [2.9–7.1]	6.1 [3.5–8.1]	3.8 [2.1–4.9]	0.010 **
PLR	203.9 [142.3–267.4]	217.9 [174.6–332.7]	156.0 [111.8–258.1]	0.010 **
C-NLR	81.4 [23.7–385.0]	131.3 [64.3–456.1]	46.3 [16.1–361.6]	0.030 *
CRP (mg/L)	18.0 [6.1–57.0]	22.0 [4.7–72.1]	14.0 [6.1–40.1]	0.177 ns
ESR (mm/h)	65.0 [44.0–82.0]	71.5 [49.0–96.8]	59.0 [28.3–67.8]	0.020 *
Albumin (g/L)	40.0 [32.5–47.8]	39.5 [29.8–47.8]	40.0 [34.5–45.4]	0.372 ns
C3 (g/L)	1.11 [1.02–1.32]	1.13 [1.02–1.35]	1.12 [1.03–1.29]	0.622 ns
C4 (g/L)	0.29 [0.21–0.34]	0.28 [0.21–0.32]	0.29 [0.20–0.35]	0.528 ns
IgG (g/L)	12.0 [10.2–15.0]	12.2 [10.3–15.3]	11.4 [9.7–14.2]	0.584 ns
IgA (g/L)	2.27 [1.83–2.62]	2.11 [1.78–2.30]	2.60 [1.92–2.88]	0.011 * (q = 0.055)
IgM (g/L)	1.20 [0.90–1.77]	1.20 [0.95–1.66]	1.15 [0.83–1.88]	0.698 ns
Organ involvement at presentation				
Pulmonary	46/73 (63%)	26 (67%)	20 (59%)	0.628 ns
Ear–nose–throat	49 (67.1%)	32 (82.1%)	17 (50.0%)	0.006 **
Epistaxis	5 (6.8%)	3 (7.7%)	2 (5.9%)	1.000 ns
Sinusitis	23 (31.5%)	13 (33.3%)	10 (29.4%)	0.803 ns
Treatment				
Intravenous methylprednisolone pulse	36 (49%)	21 (54%)	15 (44%)	0.484 ns
Cyclophosphamide-based induction	60 (82%)	34 (87%)	26 (76%)	0.358 ns
Rituximab-based induction	1 (1%)	1	0	—
Plasma exchange	16 (22%)	13 (33%)	3 (9%)	0.021 *
Acute haemodialysis	7 (10%)	5 (13%)	2 (6%)	0.438 ns
Sample obtained before any immunosuppression	73 (100%)	39 (100%)	34 (100%)	—
Clinical remission at 6 months	62/70 (88.6%)	34/39 (87%)	28/31 (90%)	1.000 ns

Data are median [IQR] or n (%). p-values by Mann–Whitney U test (continuous) or Fisher’s exact test (categorical). *p < 0.05; **p < 0.01; ***p < 0.001; ns, not significant. q denotes the Benjamini–Hochberg FDR-adjusted p-value across the five immune parameters. AZA, azathioprine; BVAS, Birmingham Vasculitis Activity Score; C-NLR, CRP-to-lymphocyte ratio; eGFR, estimated glomerular filtration rate (CKD-EPI 2021); FFS, Five-Factor Score; MMF, mycophenolate mofetil; NLR, neutrophil-to-lymphocyte ratio; PLR, platelet-to-lymphocyte ratio. Pulmonary and ENT involvement were available for all 73 patients. Six-month remission status was available for 70/73 patients (39/39 PR3-ANCA and 31/34 MPO-ANCA); three MPO-ANCA patients lacked evaluable 6-month outcome data.

Treatment exposure is reported in **Table 2** to the extent supported by the source database. Intravenous methylprednisolone pulses were administered to 36/73 patients (49%), and cyclophosphamide was used in 60/73 (82%); rituximab was recorded in 1/73 (1%). Plasma exchange was used in 16/73 patients (22%), more frequently in PR3-ANCA disease (33% vs. 9%, p = 0.021), and acute haemodialysis in 7/73 (10%). Small numbers were recorded under mycophenolate, azathioprine, and methotrexate, but the retrospective database did not permit reliable classification of these entries as induction versus maintenance therapy.

Clinical remission at 6 months was achieved in 62 of 70 patients with available outcome data (88.6%), with no significant difference between serotypes (PR3-ANCA 34/39 [87%] vs. MPO-ANCA 28/31 [90%], Fisher p = 1.000).

Baseline characteristics are presented in **Table 2**.

### 3.2 Complement and immunoglobulin profiles by ANCA serotype

Among the five immune parameters examined, IgA was the only variable to differ significantly between PR3-ANCA and MPO-ANCA patients at the nominal level (2.11 [1.78–2.30] vs. 2.60 [1.92–2.88] g/L, p = 0.011), with all values remaining within the reference interval. C3, C4, IgG, and IgM did not differ between serotypes (all p > 0.52). After Benjamini–Hochberg adjustment across the five parameters, the IgA difference did not retain significance (q = 0.055). Box-plot distributions with individual data points and reference intervals are shown in **Figure 1**.

**Figure 1:**
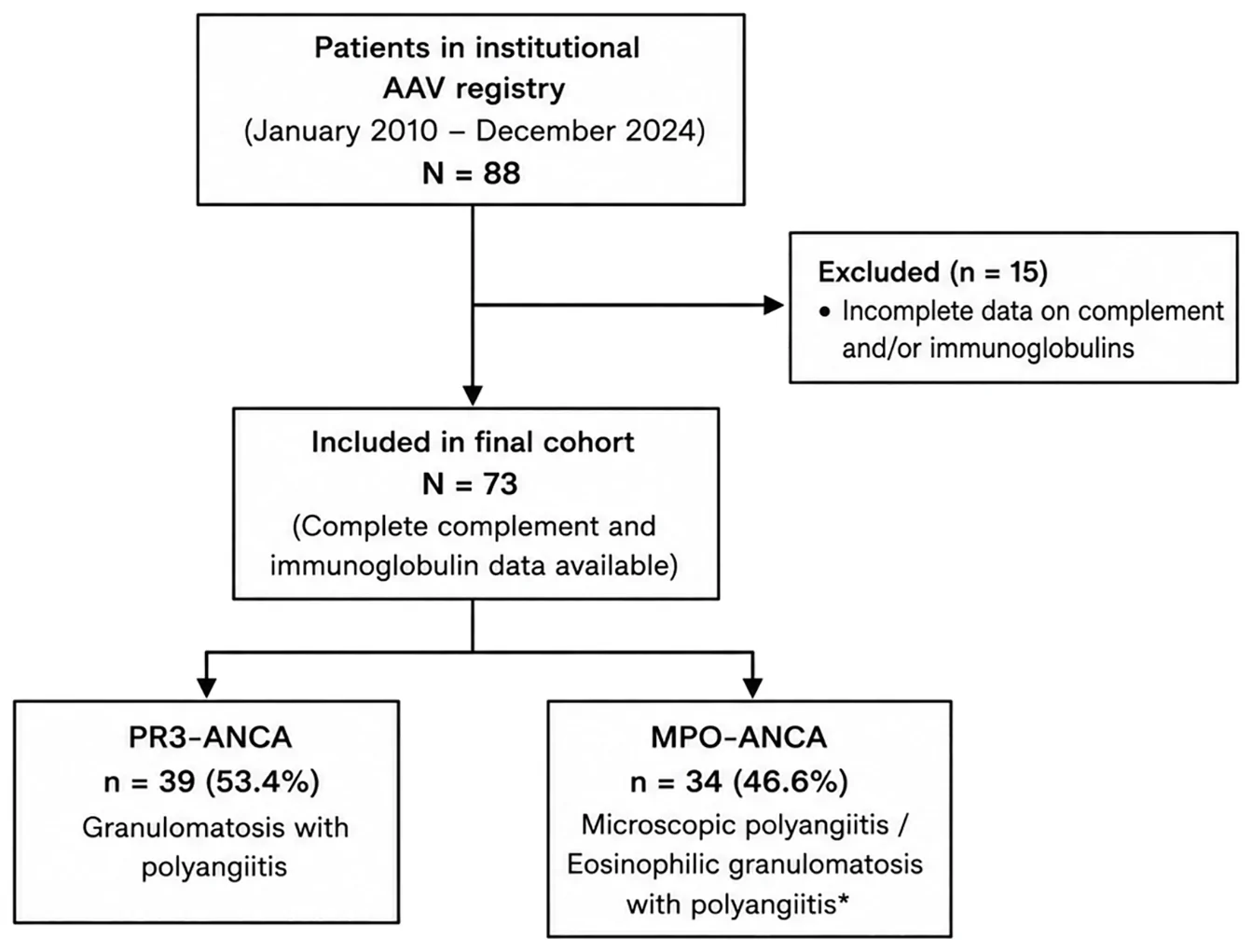
Serum complement fractions and immunoglobulin classes stratified by ANCA serotype (PR3-ANCA, n = 39; MPO-ANCA, n = 34). Box plots show median, IQR, and 1.5 × IQR whiskers; individual data points overlaid. Grey shading indicates the reference interval. *p < 0.05; ns, not significant (Mann–Whitney U test). Nominal p-values are shown; the IgA comparison did not retain significance after Benjamini–Hochberg adjustment across the five parameters (q = 0.055).

In the unadjusted comparison, IgA was higher in MPO-ANCA than PR3-ANCA disease (2.60 [1.92–2.88] vs. 2.11 [1.78–2.30] g/L; p = 0.011), but did not survive FDR correction (q = 0.055). We did not retain the previously explored multivariable log-IgA model because induction-regimen coding was too sparse and heterogeneous for a stable adjustment: 60/73 patients received cyclophosphamide-based induction, only one received rituximab, and several other immunosuppressant entries could not be reliably assigned to induction versus maintenance. Accordingly, no independent serotype effect is claimed.

### 3.3 Complement fractions and immunoglobulin classes across CKD severity groups

C3 was within the reference interval in 71/73 patients (97.3%); two patients (2.7%) had C3 below the lower reference limit of 0.90 g/L. C4 was within the reference interval in 63/73 patients (86.3%), with five values below and five above the reference interval. IgG was above its reference interval in 16/73 patients (21.9%). Neither complement fraction demonstrated a gradient across the three CKD severity groups (C3: Kruskal–Wallis (KW) p = 0.453; C4: KW p = 0.550; **Table 3**). Spearman correlations confirmed the absence of a relationship between C3 or C4 and admission eGFR (ρ = +0.033, p = 0.780 and ρ = −0.108, p = 0.362, respectively).

**Table 3:** Complement fractions and immunoglobulin classes across CKD severity groups at admission.

Parameter	eGFR < 15 (n = 27)	eGFR 15–29 (n = 15)	eGFR ≥30 (n = 31)	KW p	FDR q	*Post-hoc*
C3 (g/L)	1.17 [1.02–1.39]	1.09 [0.96–1.33]	1.09 [1.03–1.26]	0.453 ns	0.892	—
C4 (g/L)	0.30 [0.25–0.35]	0.28 [0.20–0.36]	0.28 [0.20–0.33]	0.550 ns	0.892	—
IgG (g/L)	12.6 [10.7–15.8]	11.0 [10.3–12.6]	11.7 [7.6–13.8]	0.162 ns	0.810	—
IgA (g/L)	2.14 [1.85–2.47]	2.30 [1.83–2.76]	2.43 [1.83–2.79]	0.706 ns	0.892	—
IgM (g/L)	1.20 [0.85–1.90]	1.10 [1.03–1.40]	1.20 [0.90–1.60]	0.892 ns	0.892	—
NLR	5.15 [3.12–8.53]	3.88 [2.66–5.31]	3.58 [2.52–6.68]	0.072 ns	—	—
PLR	227.7 [174.6–357.5]	154.2 [110.6–194.1]	194.1 [132.6–267.4]	0.042 *	—	< 15 vs. 15–29: p = 0.005
C-NLR	297.2 [58.2–1083.3]	32.0 [7.9–112.6]	76.2 [11.9–385.0]	0.037 *	—	< 15 vs. 15–29: p = 0.041; < 15 vs. ≥30: p = 0.024
BVAS	20.0 [14.5–21.0]	16.0 [12.0–19.0]	13.0 [12.0–17.0]	0.001 **	—	< 15 vs. ≥30: p = 0.001
FFS	2.0 [2.0–2.0]	1.0 [1.0–1.0]	1.0 [1.0–2.0]	< 0.001 ***	—	< 15 vs. 15–29 and ≥30: p < 0.001

Data are median [IQR]. KW, Kruskal–Wallis test. FDR q-values are Benjamini–Hochberg–adjusted across the five immune parameters. *Post-hoc* comparisons were performed by Mann–Whitney U test where the KW test was significant.

* p < 0.05 ** p< 0.01 *** p<0.001; ns, not significant.

Immunoglobulin classes were likewise uniformly distributed across CKD groups (all KW p > 0.16). IgG values were numerically higher at the most severe end of renal impairment without reaching significance (eGFR < 15: 12.6 [10.7–15.8] g/L; ρ vs. eGFR at admission = −0.209, p = 0.077). In contrast, C-NLR and PLR differed significantly across CKD groups (KW p = 0.037 and p = 0.042, respectively), indicating that inflammatory indices—unlike complement fractions—do track renal injury severity in this cohort. Distributions are shown in **Figure 2**.

**Figure 2:**
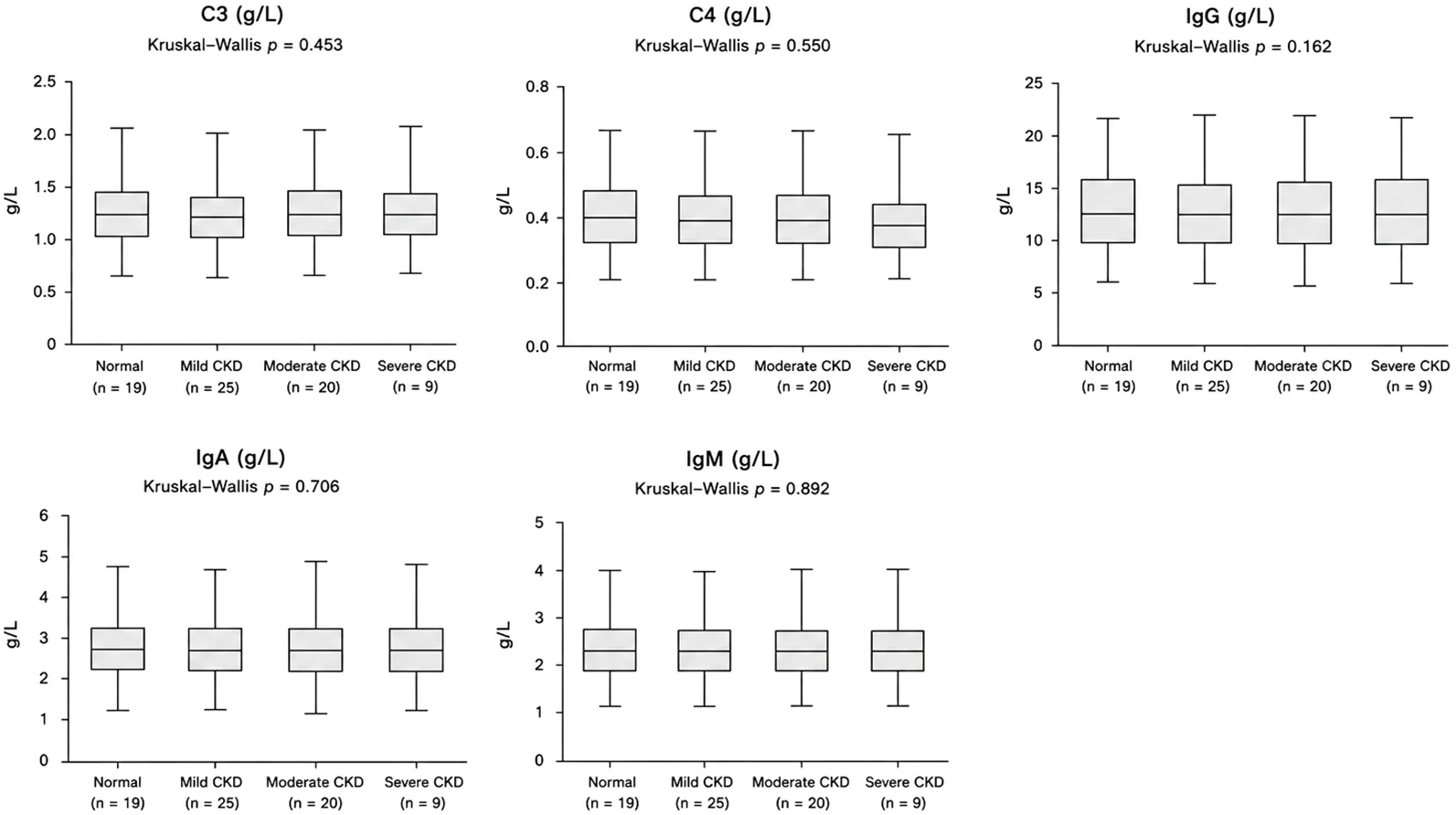
Complement fractions and immunoglobulin classes stratified by CKD severity group at admission (eGFR < 15, n = 27; 15–29, n = 15; ≥30 mL/min/1.73 m², n = 31). Box plots show median, IQR, and 1.5 × IQR whiskers, with individual data points overlaid. Grey shading: reference interval. Kruskal–Wallis p-values shown above each panel.

### 3.4 Correlations between immune parameters, disease activity and renal function

Spearman rank correlations between complement/immunoglobulin parameters and key clinical variables are presented in **Table 1** and visualized in **Figure 3**. These are unadjusted, exploratory analyses and are not corrected for multiple testing; they describe the structure of the data rather than independent associations. IgG correlated with erythrocyte sedimentation rate (ESR) (ρ = +0.40, p < 0.001), CRP (ρ = +0.26, p = 0.028), PLR (ρ = +0.24, p = 0.043), and ΔeGFR (ρ = +0.26, p = 0.037). IgA correlated with ESR (ρ = +0.31, p = 0.009). IgM correlated with CRP (ρ = +0.37, p = 0.001), C-NLR (ρ = +0.30, p = 0.014), and ΔeGFR (ρ = +0.25, p = 0.041). C3 showed an inverse correlation with NLR (ρ = −0.24, p = 0.043), and C4 correlated inversely with eGFR at 6 months (ρ = −0.25, p = 0.043).

**Figure 3:**
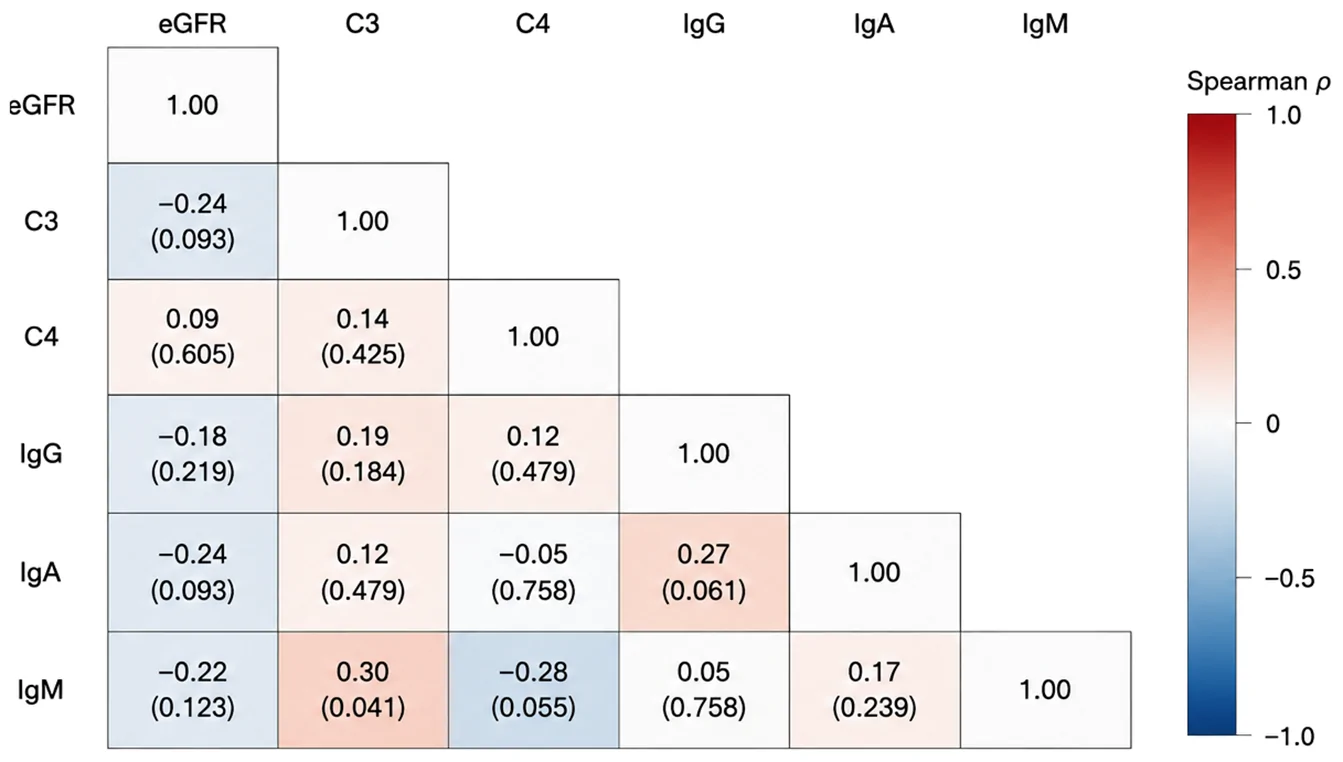
Spearman rank correlation heatmap between complement/immunoglobulin parameters (rows) and clinical variables (columns). Cell annotations show Spearman ρ and significance level. Color scale: red, positive; blue, negative. *p < 0.05; **p < 0.01; ***p < 0.001. Values are nominal and uncorrected for multiple comparisons.

### 3.5 Immune parameters and 6-month outcomes

Serum C4 at admission was higher in patients who did not achieve clinical remission at 6 months than in those who did (0.35 [0.28–0.44] vs. 0.28 [0.20–0.33] g/L, nominal p = 0.037; FDR-adjusted q = 0.184; **Table 4**). Thus, the direction of the observed association was that higher—not lower—C4 was associated with failure to achieve remission. This is the opposite of the complement-consumption framework and of published AAV studies in which lower complement, particularly C3, has been associated with adverse outcomes. ROC analysis yielded an area under the curve (AUC) of 0.729 (95% percentile-bootstrap CI 0.473–0.953) for C4 as a predictor of failure to achieve remission; the confidence interval includes 0.5, and this analysis is therefore descriptive rather than evidence of discriminative ability. The Youden-optimal cut-off was C4 > 0.31 g/L, with sensitivity 75.0% (6/8) and specificity 67.7% (42/62). Given only eight events, the percentile-bootstrap interval and the data-derived threshold are unstable; the apparent discordance between the ROC confidence interval and the Mann–Whitney p-value is considered a small-sample artefact.

**Table 4:** Complement fractions and immunoglobulin classes by clinical remission status at 6 months.

Parameter	Remission (n = 62)	Non-remission (n = 8)	Nominal p	FDR q
C3 (g/L)	1.12 [1.02–1.33]	1.14 [1.01–1.30]	0.949 ns	0.949
C4 (g/L)	0.28 [0.20–0.33]	0.35 [0.28–0.44]	0.037 *	0.184
IgG (g/L)	12.0 [10.2–15.9]	10.5 [8.4–12.4]	0.209 ns	0.348
IgA (g/L)	2.27 [1.83–2.62]	1.99 [1.53–2.25]	0.169 ns	0.348
IgM (g/L)	1.20 [0.93–1.77]	1.00 [0.88–2.05]	0.919 ns	0.949

Data are median [IQR]. p values by Mann–Whitney U test; q values are Benjamini–Hochberg–adjusted across the five parameters. C4 AUC = 0.729 (95% bootstrap CI 0.473–0.953; the interval includes 0.5). Youden-optimal cut-off C4 > 0.31 g/L: sensitivity 75.0% (6/8), specificity 67.7% (42/62). With only eight non-remission events, the percentile-bootstrap ROC interval is unstable; its discordance with the nominal Mann–Whitney p-value is interpreted as a small-sample artefact.

In a partial Spearman correlation adjusted for ANCA serotype, the association between admission C4 and remission status was ρ_partial = −0.257 (p = 0.033; n = 70). The association was attenuated and no longer significant when treatment-related covariates were included. Excluding the 16 patients exposed to plasma exchange, C4 was 0.27 [0.20–0.31] g/L in remission versus 0.31 [0.21–0.49] g/L in non-remission (p = 0.300). Restricted to patients receiving cyclophosphamide, corresponding C4 values were 0.28 [0.20–0.32] versus 0.31 [0.21–0.33] g/L (p = 0.563).

IgG and IgM correlated positively with ΔeGFR (IgG: ρ = +0.26, p = 0.037; IgM: ρ = +0.25, p = 0.041). These are unadjusted rank correlations of modest magnitude; the present data cannot establish that either immunoglobulin contributes information independent of conventional inflammatory indices. **Figure 4** presents C4 and IgG distributions by remission status with the C4 ROC curve. Key scatter plots are shown in **Figure 5**.

**Figure 4:**
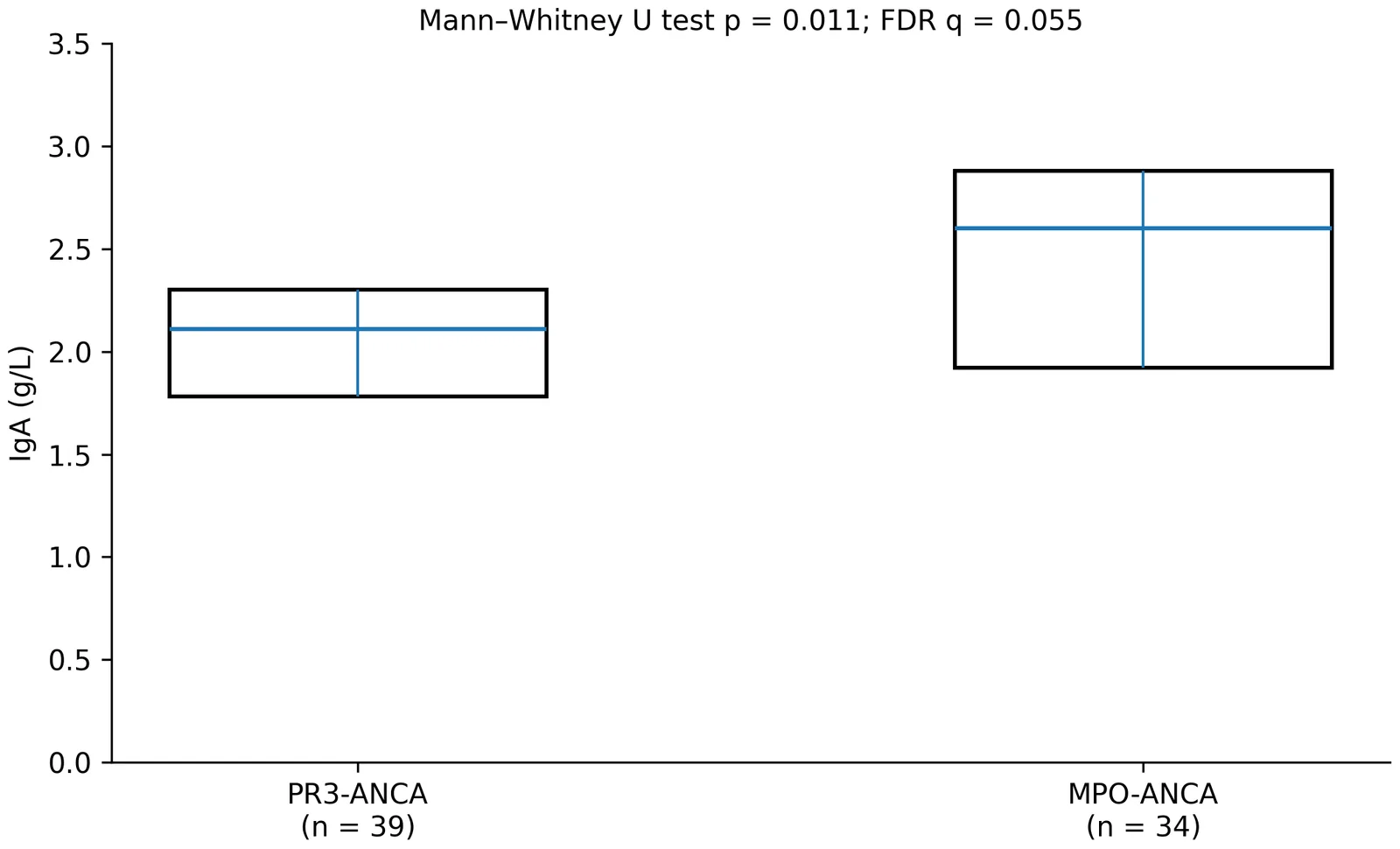
Box plot comparing IgA concentrations in grams per liter between PR3-ANCA (n equals 39) and MPO-ANCA (n equals 34) groups, with a Mann-Whitney U test p-value of 0.011 and FDR q-value of 0.05.

**Figure 5:**
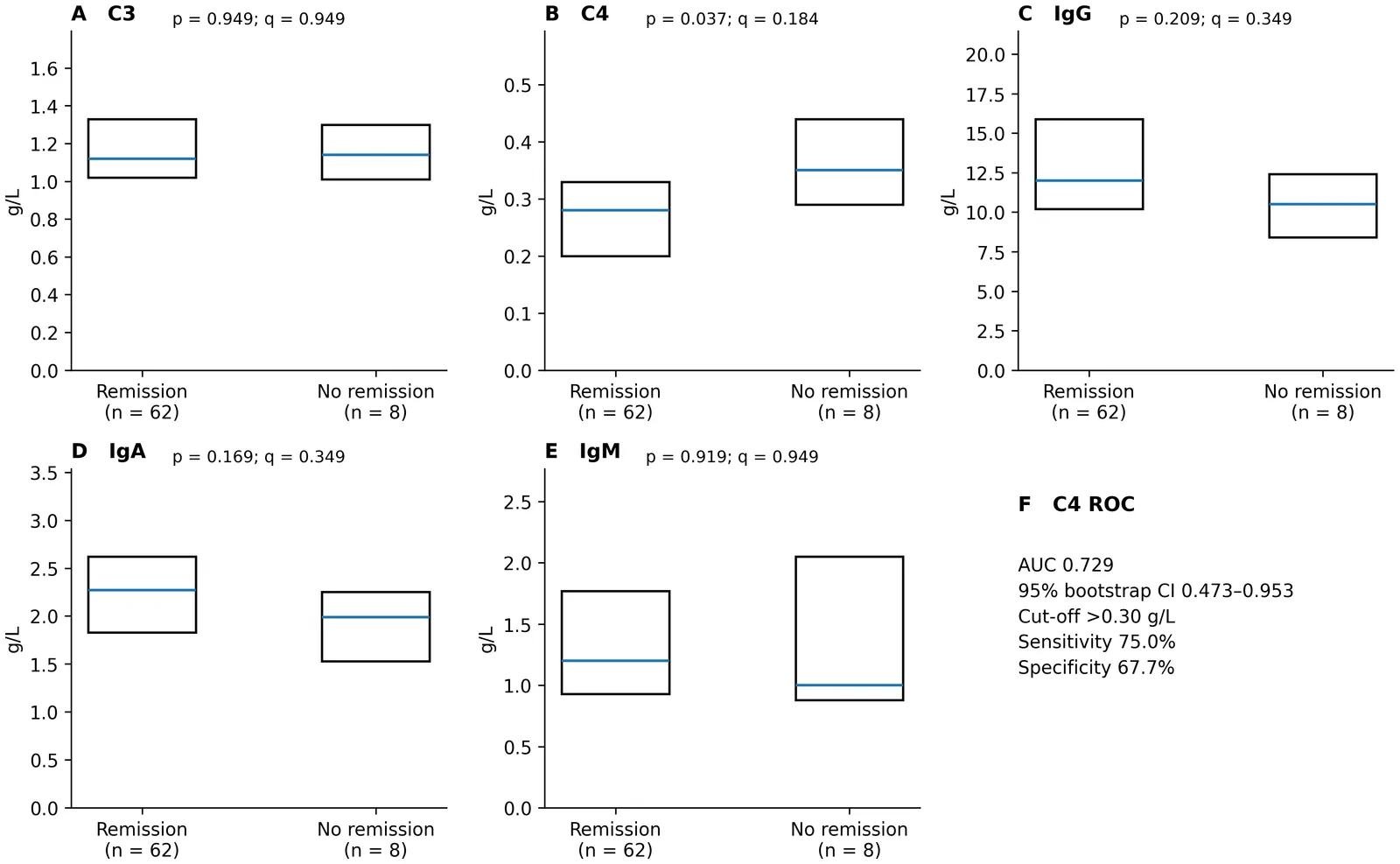
Five box plots labeled **(A–E)** compare the concentrations of C3, C4, IgG, IgA, and IgM between remission and no remission groups, while panel F summarizes C4 ROC analysis with an AUC of 0.729, cut-off above 0.30 g/L, sensitivity 75.0 percent, and specificity 67.7 percent.

## 4 Discussion

In this single-center cohort of AAV with renal involvement, we characterized complement fractions together with immunoglobulin class profiles, stratified by ANCA serotype, CKD severity, and 6-month outcome. The principal observation is that overt complement consumption was uncommon—97.3% of patients had C3 and 86.3% had C4 within the reference interval—and that neither fraction showed a gradient across CKD severity groups, whereas inflammatory indices measured in the same patients did. In this respect, AAV differs from lupus nephritis, in which depression of C3 and C4 is an established correlate of immune complex–mediated glomerular injury (5, 6).

This observation must be set alongside a consistent body of evidence that serum C3 at diagnosis does carry prognostic weight in AAV, and our original framing did not engage adequately with that literature. Augusto et al. found that, although C3 remained within the reference interval throughout their cohort of 45 patients, those below the cohort median had significantly poorer patient and death-censored renal survival, with lower C3 associated with a greater proportion of glomeruli showing cellular crescents (7). Crnogorac et al. studied 75 patients with renal AAV using the same reference interval as our laboratory (0.90–1.80 g/L) and found that C3 below 0.90 g/L at diagnosis predicted end-stage kidney disease and death (8); Villacorta et al. reported comparable associations with renal and global outcomes (9), as did Choi et al. in 139 immunosuppression-naïve patients (10). Our findings are not in conflict with these reports. What they indicate is where the signal does—and does not—reside. C3 did not fall as eGFR fell, and did not stratify across CKD stage; yet it varied inversely with neutrophil activation as captured by the NLR. We therefore revise the interpretation offered in the original submission: rather than concluding that C3 and C4 are uninformative in AAV, the defensible conclusion is that they are not markers of the extent of renal injury, while relative variation within the reference interval may still index complement-pathway engagement—a distinction that matters clinically, because it means a normal C3 in a patient with AAV cannot be read as reassurance about the severity of renal disease, and a value in the lower part of the reference range should not be dismissed as normal.

One quantitative discrepancy deserves explicit comment. Frank hypocomplementaemia was uncommon in our cohort, whereas approximately one-fifth of patients in the cohorts of Crnogorac et al. and Choi et al. had C3 below the lower reference limit (8, 10). Because all complement measurements in our cohort were obtained before immunosuppressive treatment and before plasma exchange, treatment exposure cannot explain this difference. Potential explanations include differences in referral patterns, assay characteristics, disease phenotype, and cohort composition. This heterogeneity limits direct comparison of absolute complement concentrations across AAV cohorts.

The inverse correlation between C3 and NLR (ρ = −0.24, p = 0.043) is consistent with experimental evidence that NET formation drives complement activation and endothelial injury (4). It should nevertheless be interpreted with restraint: the coefficient is weak, the p value is nominal and would not survive correction across the correlation matrix, the NLR is a crude and non-specific surrogate for NET formation, and no NET marker (for example, MPO–DNA complexes or citrullinated histone H3) or complement activation product (C3a, C5a, Bb, sC5b-9) was measured. We therefore present this as a hypothesis-generating observation compatible with—rather than evidence for—the NET–complement axis operating in patients, and we have removed the stronger claim made in the original submission.

The higher serum IgA in MPO-ANCA than in PR3-ANCA patients was the only nominal between-serotype difference among the five immune parameters examined, but it requires caution. First, it did not survive Benjamini–Hochberg adjustment (q = 0.055). Second, every individual value lay within the laboratory reference interval, and the 0.49 g/L difference in medians has no established interpretation at the individual-patient level. Third, we did not retain the previously explored multivariable log-IgA model because induction-regimen coding was too sparse and heterogeneous for stable adjustment: 60/73 patients received cyclophosphamide-based induction, only one received rituximab, and several other immunosuppressant entries could not be reliably classified as induction versus maintenance therapy. Accordingly, no independent serotype effect is claimed. Finally, clinicopathological phenotype and ANCA serotype were highly concordant, so their independent contributions cannot be separated in this cohort. We therefore present the IgA observation as hypothesis-generating and make no claim of diagnostic utility.

If a genuine serotype difference is confirmed, mucosal immune biology offers plausible mechanisms. IgA-class ANCA have been reported in a substantial minority of patients with AAV; IgA can prime and activate neutrophils through FcαRI, a pathway capable of inducing NET formation; and glomerular IgA deposition in MPO-ANCA glomerulonephritis has been associated with vascular and granuloma-like lesions (22). None of these mechanisms was measured here, and total serum IgA concentration is a poor surrogate for any of them (23). It is also relevant that the serotype-specific humoral differences convincingly demonstrated in AAV to date have been qualitative rather than quantitative—differing IgG Fc galactosylation and sialylation profiles, which track disease activity in PR3-ANCA but not in MPO-ANCA disease (15). If genuine serotype-specific immunoglobulin signatures exist in AAV, they are more likely to be found in glycosylation, subclass distribution, and antigen specificity than in total class concentrations. We therefore present the IgA observation as hypothesis-generating, and regard IgA subclass quantification, IgA-ANCA testing, and structured mucosal phenotyping in a prospective cohort as the appropriate next step rather than any change in current practice.

IgG was the immunoglobulin class most consistently associated with inflammatory burden and renal recovery trajectory. Its correlations with ESR and CRP are compatible with IgG reflecting the humoral inflammatory milieu in active AAV, while the positive correlation with ΔeGFR is an exploratory association with renal recovery. These are unadjusted rank correlations and cannot establish independent or incremental prognostic value. The claim that IgG and C4 carry prognostic information “beyond inflammatory indices”, made in the original abstract and conclusions, was not supported by the analyses presented and has been removed. Establishing incremental prognostic value would require multivariable modelling with an adequate number of events, which this cohort cannot support.

The association between higher admission C4 and failure to achieve remission requires the same restraint. Importantly, its direction is counter to the complement-consumption framework: in our data, higher C4 was associated with non-remission, whereas the published AAV outcome literature has generally linked lower complement—most consistently lower C3—with adverse outcomes. We therefore do not interpret this C4 finding as evidence of greater complement consumption or as confirmation of the established C3 prognostic signal. It rests on only eight events and did not survive FDR adjustment (q = 0.184). The ROC AUC was 0.729, but its 95% percentile-bootstrap confidence interval included 0.5 (0.473–0.953), and the Youden-derived operating characteristics (sensitivity 6/8 [75.0%]; specificity 42/62 [67.7%]) are unstable. The apparent discordance between the Mann–Whitney p-value and the bootstrap ROC interval is most plausibly a small-sample artefact. The association was not retained after exclusion of plasma-exchange patients (p = 0.300) or restriction to cyclophosphamide-treated patients (p = 0.563). With only eight events, a multivariable treatment-adjusted logistic model would be unstable. We therefore cannot distinguish an independent prognostic effect of C4 from chance, confounding by treatment allocation, or disease phenotype, and present this observation solely as an exploratory signal.

Treatment exposure warrants separate comment because it is an important determinant of clinical outcome. All complement and immunoglobulin samples in this study were obtained before initiation of immunosuppressive treatment, and none were obtained after plasma exchange had begun; therefore, the baseline immune measurements were not altered by preceding treatment. Treatment nevertheless remains relevant to remission and renal recovery, which is why treatment-restricted sensitivity analyses were performed for the C4–remission association.

A further limitation of the comparison made throughout this manuscript should be stated plainly: no lupus nephritis patients were studied. The contrast with LN rests entirely on published data rather than on a contemporaneous comparator group measured in the same laboratory with the same assays, and the language throughout has been moderated accordingly.

Other limitations include the retrospective single-center design; the moderate sample size (n = 73) and small number of non-remission events (n = 8); the exploratory nature of the analyses and the number of comparisons performed, with the two headline findings not surviving FDR adjustment; incomplete retrospective documentation of several extrarenal manifestations and maintenance treatment; the absence of complement pathway–specific biomarkers (C3a, C5a, Bb, sC5b-9, factor H) and of tissue complement staining; the absence of B-cell subpopulation and immunoglobulin subclass analysis; a single measurement time point, precluding any statement about temporal relationships; and the absence of systematic kidney biopsy classification. Serial complement and immunoglobulin measurement at onset, during induction, and at remission or relapse, in a multicentre cohort with prospectively recorded treatment and organ involvement, would be required to move any of these observations beyond hypothesis.

## 5 Conclusions

In ANCA-associated vasculitis with renal involvement, serum complement fractions were preserved across the full spectrum of renal injury severity and showed no gradient with declining eGFR, consistent with the pauci-immune pathogenesis of AAV glomerulonephritis and contrasting with the pattern described in lupus nephritis. C3 and C4 should not be used as markers of the extent of renal injury in AAV; this is compatible with, rather than contrary to, published evidence that relatively lower C3 within the reference interval predicts adverse renal outcome, and the two observations together locate the complement signal in AAV in subtle within-range variation rather than in measurable consumption. The inverse correlation between C3 and NLR is compatible with an active NET–complement axis but does not establish one. Higher IgA in MPO-ANCA than PR3-ANCA disease and higher admission C4 in patients who failed to achieve remission are exploratory observations that did not survive correction for multiple comparisons. No adjusted IgA model was retained because induction-regimen coding was too sparse and heterogeneous for stable treatment adjustment, whereas the small number of non-remission events precluded reliable multivariable adjustment of the C4 association. These findings are reported as hypotheses for prospective testing and not as biomarkers for clinical use. Prospective, treatment-adjusted, multicentre studies incorporating complement activation products and immunoglobulin subclass analysis are required before complement and immunoglobulin profiling can be recommended as part of routine immune phenotyping in AAV.

## Data Availability

The raw data supporting the conclusions of this article will be made available by the authors, without undue reservation.
